# Evaluation of e-health (Seha) application: a cross-sectional study in Saudi Arabia

**DOI:** 10.1186/s12911-021-01437-6

**Published:** 2021-03-18

**Authors:** Abeer Alharbi, Joharah Alzuwaed, Hind Qasem

**Affiliations:** 1 Health Administration Department, Business Administration School, King Saud University, Riyad, Saudi Arabia; 2 Biostatistics Department, Inaya Medical Colleges, Riyadh, Saudi Arabia

**Keywords:** e-Health, Telemedicine, Access, Satisfaction, Efficiency, Health care delivery, Saudi Arabia

## Abstract

**Background:**

The Ministry of Health in Saudi Arabia is expanding the country’s telemedicine services by using advanced technology in health services. In doing so, an e-health application (app), Seha, was introduced in 2018 that allows individuals to have face-to-face visual medical consultations with their doctors on their smartphones.

**Objective:**

This study evaluated the effectiveness of the app in improving healthcare delivery by ensuring patient satisfaction with the care given, increasing access to care, and improving efficiency in the healthcare system.

**Methods:**

A cross-sectional study design was used to assess the perceptions of users of the Seha app and non-users who continued with traditional health services. The data were collected using an online survey via Google Forms between June 2020 and September 2020. Independent t tests and chi-square (*χ*^2^) tests were conducted to answer the research questions.

**Results:**

There was a significant difference between users and non-users in terms of ease of access to health services (t =  − 9.38, *p* < 0.05), with app users having a higher mean score (4.19 ± 0.91) than non-users (3.41 ± 1.00); satisfaction with health services (t =  − 6.33, *p* < 0.05), with users having a higher mean score (3.96 ± 0.91) than non-users (3.45 ± 0.94); and efficiency (only one visit needed for treatment) (t =  − 3.20, *p* < 0.05), with users having a higher mean score (3.71 ± 0.93) than non-users (3.45 ± 0.93). There were significant associations between the use of the Seha app and age (*χ*^2^ = 8.79, *p* < 0.05), gender (*χ*^2^ = 22.19, *p* < 0.05), region (*χ*^2^ = 19.74, *p* < 0.05), and occupation (*χ*^2^ = 22.05, *p* < 0.05). There were significant relationships between the three items (on access, satisfaction, and efficiency) and experiencing technical issues (t = 4.47, t = 8.11, and t = 3.24, respectively, *p* < 0.05), with users who faced technical problems having significantly lower mean scores for all three items.

**Conclusion:**

This study provided evidence that the Seha app improved the delivery of healthcare in Saudi Arabia. Users of the app had a better health experience in terms of their perceived ease of access to healthcare services; their satisfaction with healthcare services; and the efficiency of the system, measured by the number of required doctor visits. Other factors that appeared to influence the use of the app included age, gender, usual source of care, and technical difficulties.

**Supplementary Information:**

The online version contains supplementary material available at 10.1186/s12911-021-01437-6.

## Background

Advanced information and communication technologies, such as smartphones, are revolutionizing the delivery of healthcare. Electronic consultations, or e-consultations, via mobile phones offer increased care accessibility and improve patients’ satisfaction with health services. The quality of virtual consultations may be comparable to that of traditional face-to-face office visits, with the additional benefit of enhanced access to care [1–9]. Several health systems have started programs to provide healthcare directly to patients through digital applications (apps). For example, in the U.S., *American Well*, *Teladoc*, and *Doctor on Demand* provide e-consultations, including for diagnostics and treatments. In the U.K., *GP at Hand* provides video consultations for patients in the city of London, and the consultations will be available for every patient in England by 2021. In Saudi Arabia, the Ministry of Health (MoH) is expanding the country’s telemedicine services by using advanced technology in health services. In doing so, an e-health app called Seha was introduced in 2018 to allow individuals to have face-to-face visual medical consultations with their doctors on their smartphones. The app is designed to enable audio–video communication, as users can login into the app, communicate directly with a specialist and have their cases diagnosed through the app. Hence, a specialist answers users' inquiries, conducts the needed medical consultation and provides the required medical procedure.

The healthcare system in Saudi Arabia is a national system in which health services are provided through the government free of charge under three main sectors: the MoH network of hospitals and primary healthcare centres, through which health services are distributed throughout the country; other governmental institutions (military or university hospitals); and the private sector. The MoH is the largest provider of healthcare services in Saudi Arabia and is responsible for over 60% of healthcare services, with the remaining 40% provided by other governmental and private facilities [10]. Given that health services are provided free of charge, patients often endure long waits before seeing specialists. The demand for health services is increasing, but the resources are still not adequate [11, 12]. As a result, health service users are dissatisfied with the provision of healthcare in terms of its quality and waiting times [13]. Saudi Vision 2030, an initiative established to reduce Saudi Arabia’s dependence on oil by diversifying its economy, also seeks to improve and develop all public services, including health care services. The vision has positioned e-health as a primary transformational enabler of high-quality and patient-centric care. E-health efforts in Saudi Arabia have been found to be efficient in reducing the time cost and amount of effort required to provide care for patients [14]. The development of the Seha mobile health app was driven by the expectation that the adoption of telemedicine will provide sustainable solutions and improve access and satisfaction with healthcare services in Saudi Arabia.

The literature shows that online consultations improve the delivery of healthcare in terms of access and satisfaction [1–8]. However, some studies have found that online consultations may be suitable for simple problems not requiring physical examination but less ‘information rich’ than face-to-face consultations [5, 15, 16]. Furthermore, technical issues have been found to be a barrier to the use of the technology [15]. In Saudi Arabia, consultations via the Seha app constituted 2% of primary care visits in 2018 [17]. A total of 1,877,440 e-consultations were conducted using the Seha app in the first half of 2020 [18]. The patients who used the app were generally satisfied with the service [17]. However, recent studies have shown that the general public in Saudi Arabia still lacks knowledge and experience with the Seha app and is reluctant to use it due to a lack of trust in e-health apps [19, 20]. Therefore, there is a crucial need for the development of more effective telemedicine apps. This aim could be achieved by evaluating patients’ perceived benefits of using the Seha app and by considering the demographic, organizational, and technical factors affecting the use of the app. Although the number of e-consultations via the Seha app is increasing, the literature is still limited in terms of evidence of the effectiveness of the Seha app in improving the delivery of healthcare in Saudi Arabia and reducing the need for more traditional health providers, who are in short supply.

This study aimed to assess the potential impact of e-health apps on population health in Saudi Arabia through the use of the Seha diagnostic mobile medical app. The overall goal of this study was to evaluate the effectiveness of the Seha app in improving healthcare delivery and population health by ensuring patient satisfaction with the care given, increasing access to care, and improving efficiency in the healthcare system. The study also aimed to understand the potential factors contributing to the use of the Seha app and determine whether there are technical issues affecting access, satisfaction, and efficiency. Specifically, the study examined the following research questions:

1. Are there significant differences between users and non-users of the Seha app in terms of (a) their perceived ease of access to health services, (b) their satisfaction with health services, and (c) the efficiency of the health services (i.e., only one visit was needed for treatment)?
2. Are there significant associations between the use of the Seha app and users’ (a) demographic characteristics and (b) usual source of care?
3. Are there significant associations between Seha user experience of technical issues and access, satisfaction, and efficiency?

## Methods

### Study design

We used a cross-sectional study design to assess the perceptions of the users of the Seha app and non-users who continued with traditional health services. This design allowed us to compare different groups and assess the relationship between the app and the perceived ease of accessing the health system; user satisfaction with the health system; and the efficiency of the health system, measured by the number of visits needed for treatment.

### Data collection and instrument

The data were collected using an online survey via Google Forms between June 2020 and September 2020. We crafted the survey constructs after conducting an extensive literature review of relevant research constructs that had previously been validated in other studies [21–24]. The first part of the survey collected information on the demographic characteristics of the participants, including their gender, age, marital status, region, education, income, occupation, and usual source of care. Then, the survey asked the participants if they had ever used the Seha app. Based on their ‘Yes’ or ‘No’ answer, the participants were divided into two groups to answer the remainder of the questionnaire. Those answering ‘Yes’ were directed to the first part of the instrument with questions addressed to Seha users; those answering ‘No’ were guided to the second part for non-users. For the Seha users, the survey asked about the perceived ease of accessing the health system, satisfaction with the health system, and the efficiency of the system in terms of the number of visits they needed to make for treatment while using the Seha app. Additionally, the survey asked the Seha users whether they faced technical difficulties while using the app. The respondents rated the following statements on a 5-point Likert scale ranging from 1 = strongly disagree to 5 = strongly agree:

- The (Seha) app facilitated access to health services.
- I am satisfied with the health services provided through the Seha app.
- When I used the Seha app, one visit (consultation) was required to deal with my condition.

For the non-users, the survey asked about the perceived ease of accessing the health system, satisfaction with the health system, and the number of clinic visits needed for treatment while using traditional health services. The survey also asked the non-users whether they were aware of the existence of the Seha app. The non-users rated the following statement on the same 5-point Likert scale as the users:

- Access to traditional health services is easy.
- I am satisfied with traditional health services.
- When I used traditional health services, only one visit (consultation) was required to deal with my condition.

The instrument’s validity and reliability were tested, and the results showed that it was both reliable and valid. The questions in the first part of the survey addressed to the Seha users had a Cronbach’s alpha = 0.70, which indicated that this part of the questionnaire was highly reliable. All three scale items (on access, satisfaction, and efficiency) showed a significant correlation with the total score (*p* ≤ 0.05), which indicated that this part of the questionnaire was valid. Similarly, the survey questions addressed to the non-users had a Cronbach’s alpha = 0.88, indicating a high level of reliability. The validity of all three scale items (on access, satisfaction, and efficiency) was confirmed, as they showed significant correlations with the total score (*p* ≤ 0.05). Institutional Review Bord (IRB) approval for the study was obtained from King Saud University (KSU), reference number KSU-KSU-HE-19-468.

### Statistical analysis

The data were analysed using SPSS version 23.0. The frequencies, percentages, means and standard deviations were computed for the research variables and the demographic factors. Independent t tests and chi-square (*χ*^2^) tests were conducted to test the relationships between variables to answer the research questions. A *p*-value of less than 0.05 was considered to be statistically significant.

## Results

### Demographics

A detailed description of the demographics of the sample of 528 participants (users and non-users of the Seha app) is shown in Table 1.

**Table 1:** Demographic characteristics by group (Seha app users and non-users) (N = 528)

Characteristic	Seha app users (N = 249)		Seha app non-users (N = 279)		All (N = 528) χ^2^ = 0.17, *p* > 0.05	
	N	%	N	%	N	%
*Gender*						
Male	124	49.8	83	29.7	207	39.2
Female	125	50.2	196	70.3	321	60.8
*Age*						
18–29	61	24.5	61	21.9	122	23.1
30–49	163	65.5	164	58.8	327	61.9
> 50	25	10.0	54	19.4	79	15.0
*Marital status*						
Single	61	24.5	89	31.9	150	28.4
Married	188	75.5	190	68.1	378	71.6
*Region*						
Central	173	69.5	161	57.7	334	63.3
Eastern	9	3.6	19	6.8	28	5.3
Western	26	10.4	65	23.3	91	17.2
Southern	6	2.4	7	2.5	15	2.8
Northern	35	14.1	27	9.7	60	11.4
*Education*						
High school	36	15.6	44	17.1	80	16.4
Diploma	21	9.1	30	11.7	51	10.5
Bachelor’s	114	49.4	132	51.4	246	50.4
Master’s	52	22.5	42	16.3	94	19.3
PhD	8	3.5	9	3.5	17	3.5
*Income*						
< 5000 SAR	69	27.7	88	31.5	157	29.7
5001–10,000 SAR	62	24.9	82	29.4	144	27.3
10,001–30,000 SAR	110	44.2	100	35.8	210	39.8
> 30,000 SAR	8	3.2	9	3.2	17	3.2
*Occupation*						
Public employee	145	58.2	118	42.3	263	49.8
Private employee	21	8.4	60	21.5	81	15.3
Unemployed	67	26.9	85	30.5	152	28.8
Student	16	6.4	16	5.7	32	6.1

The distribution of the sample between the two groups showed a larger number of non-users (52.8%) than users (47.1%), but the difference in the group sizes was not statistically significant (χ^2^ = 0.17, *p* > 0.05). Approximately 60.8% of the sample was female, and with regard to age, most of the participants were within the 30–49 age group (61.9%), with 23.1% in the 18–29-year-old group and 15% in the > 50-year-old group. The majority of the participants (71.6%) were married and lived in the central region (63.3%). Approximately half of the participants (50.4%) held a bachelor’s degree, and 22.8% had a master’s degree or PhD, with the rest being high school graduates or having other types of diplomas. With regard to income, slightly more than one-third of the sample (39.8%) earned between 10,001 and 30,000 SAR, with the rest earning less than 5000 SAR (29.7%) or between 5001 and 10,000 SAR (27.3%). Nearly half of the participants (49.8%) were public employees, 28.8% were unemployed, 15.3% were private employees, and 6.1% were students. Additional file 1: Appendix 1 shows the utilization patterns of traditional health services among the non-users and their level of awareness of the app. Approximately 56% of the non-users were aware of the existence of the app. With regard to the utilization of traditional health services, approximately two-thirds of the sample (68.8%) used outpatient clinics at private hospitals, 50.2% used the emergency department, 53.4% used outpatient clinics in a public hospital, and 41.6% used primary health care centres.

### Access, satisfaction, and efficiency

An independent t test was conducted to answer the research first question, i.e., *Are there significant differences between users and non-users of the Seha app in terms of (a) their perceived ease of access to health services, (b) their satisfaction with health services and (c) the efficiency of health services (i.e., only one visit needed for treatment)?* The data are shown in Table 2.

**Table 2:** Independent t test (Seha app users vs non-users) (N = 528)

Factor	Seha app	M	SD	t	*p*
Access	Non-user	3.41	1.00	− 9.38**	< 0.000
	User	4.19	0.91		
Satisfaction	Non-user	3.45	0.94	− 6.33**	< 0.000
	User	3.96	0.91		
Efficiency (only one visit needed)	Non-user	3.45	0.93	− 3.20**	< 0.000
	User	3.71	0.93		

**p < 0.01; *p < 0.05

As shown in Table 2, there was a significant difference between the users and non-users in terms of ease of access to health services (t =  − 9.38, *p* < 0.05), with the users of the app having a higher mean score (4.19 ± 0.91) than the non-users (3.41 ± 1.00). The same result was found for satisfaction with health services (t =  − 6.33, *p* < 0.05), with the app users having a higher mean score (3.96 ± 0.91) than the non-users (3.45 ± 0.94). Similarly, there was a significant difference between the two groups in terms of efficiency (i.e., only one visit needed for treatment) (t =  − 3.20, *p* < 0.05), with the users of the app having a higher mean score (3.71 ± 0.93) than the non-users (3.45 ± 0.93). These results indicated that the level of access, satisfaction and efficiency were higher for the users of the Seha app.

### Seha app use: demographic factors and usual source of care

A chi-square (*χ*^2^) test was conducted to answer the second research question, i.e., *Are there significant associations between the use of the Seha app and users’ (a) demographic characteristics and (b) usual source of care?*

As shown in Table 3, there was a significant association between gender and the use of the Seha app (*χ*^2^ = 22.19, *p* < 0.05). The results revealed that males (59.9%) used the app more often than females (38.9%). There was a significant relationship between age and the use of the Seha app (*χ*^2^ = 8.79, *p* < 0.05). Usurpingly, older participants (68.4% of those over 50 years old) used the app less than the younger population. In addition, there was a significant relationship between use of the Seha app and region of residence (*χ*^2^ = 19.74, *p* < 0.05), with the participants from the northern region showing the highest usage (55%) and those from the western area using the app the least (28.6%). The analysis further showed a significant relationship between occupation and the use of the Seha app (*χ*^2^ = 22.05, *p* < 0.05), with public employees (55.1%) using the app the most. The rest of the findings regarding the demographic factors did not show any significant associations with the use of the Seha app (*p* > 0.05).

**Table 3:** The relationship between demographic characteristics and the use of the Seha app

Factor	User	Non-user	Total	X^2^/*p*
*Gender*				
Male	124	83	207	22.19**/< 0.000
	59.90%	40.10%	100%	
Female	125	196	321	
	38.90%	61.10%	100%	
*Age*				
18–29	61	61	122	8.79*/0.011
	50.00%	50.00%	100%	
30–49	163	164	327	
	49.80%	50.20%	100%	
> 50	25	54	79	
	31.60%	68.40%	100%	
*Marital status*				
Single	61	89	150	*3.54/0.06*
	40.70%	59.30%	100%	
Married	188	190	378	
	49.70%	50.30%	100%	
*Region*				
Central	173	161	334	19.74**/0.001
	51.80%	48.20%	100%	
Eastern	9	19	28	
	32.10%	67.90%	100.00%	
Western	26	65	91	
	28.60%	71.40%	100%	
Southern	8	7	15	
	53.30%	46.70%	100%	
Northern	33	27	60	
	55.00%	45.00%	100%	
*Education*				
Secondary school	36	44	80	*3.45/0.49*
	45.00%	55.00%	100%	
Diploma	21	30	51	
	41.20%	58.80%	100%	
Bachelor’s	114	132	246	
	46.30%	53.70%	100%	
Master’s	52	42	94	
	55.30%	44.70%	100%	
PhD	8	9	17	
	47.10%	52.90%	100%	
*Income*				
< 5000 SAR	69	88	157	*3.92/0.27*
	43.90%	56.10%	100%	
5001–10,000 SAR	62	82	144	
	43.10%	56.90%	100%	
10,001–30,000 SAR	110	100	210	
	52.40%	47.60%	100%	
> 30,000 SAR	8	9	17	
	47.10%	52.90%	100%	
*Occupation*				
Public employees	145	118	263	22.05**/< 0.000
	55.10%	44.90%	100%	
Private employees	21	60	81	
	25.90%	74.10%	100%	
Unemployed	67	85	152	
	44.10%	55.90%	100%	
Students	16	16	32	
	50.00%	50.00%	100%	

**p < 0.01; *p < 0.05

In terms of the usual source of care, Table 4 shows significant relationship between MoH facilities and military health facilities as the usual sources of care and the use of the Seha app (X^2^ = 26.08 and X^2^ = 3.70, respectively, *p* < 0.05). The participants who did not use MoH facilities as their usual source of care (70.1%) used the Seha app less often than the other participants, while those who identified military health facilities as their usual source of care used the Seha app more often (59%). There was no significant difference in Seha app use between the participants who reported private facilities as their usual source of care and those who did not (*p* > 0.05).

**Table 4:** The relationship between the type of health coverage and use of the Seha app

Usual source of care	User	Non-user	Total	*X*^*2*^/*p*
*Ministry of Health*				
No	46	108	154	26.08**/< 0.000
	29.9%	70.1%	100%	
Yes	203	171	374	
	54.3%	45.7%	100%	
*Military*				
No	150	210	360	13.70**/< 0.000
	41.7%	58.3%	100%	
Yes	99	69	168	
	58.9%	41.1%	100%	
*Private*				
No	55	58	113	*0.13/0.70*
	48.7%	51.3%	100%	
Yes	194	221	415	
	46.7%	53.3%	100%	

**p < 0.01; *p < 0.05

### Technical issues

An independent t test was conducted to answer the third research question, i.e., *Are there significant associations between Seha user experience of technical issues and access, satisfaction, and efficiency?* As shown in Table 5, there were significant relationships between the three items (on access, satisfaction, and efficiency) and the experience of technical issues when users used the app for the first time (t = 4.47, t = 8.11, and t = 3.24, respectively, *p* < 0.05). Users who faced technical problems when using the app for the first time had significantly lower mean scores for access, satisfaction, and efficiency. In addition, having continuous technical issues was significantly associated with access and satisfaction (t = 2.07 and t = 7.92, respectively, *p* < 0.05). Users who faced continuous technical issues with the app had significantly lower mean scores for access and satisfaction than those who did not, but having continuous technical issues showed no significant effect on efficiency in terms of the number of visits needed.

**Table 5:** The relationship between the mean scores on the three items (on access, satisfaction, and number of visits) and facing technical issues in the use of the app (N = 249)

Technical issues	Factor		M	SD	t	*p*
Technical problems when using the app for the first time	Access	No	4.33	0.75	*t* = 4.47**	< 0.000
		Yes	3.75	1.20		
	Satisfaction	No	4.20	0.74	*t* = 8.11**	< 0.000
		Yes	3.22	1.03		
	Efficiency (only one visit needed)	No	3.82	0.90	*t* = 3.24**	0.001
		Yes	3.38	0.94		
Continuous technical problems	Access	No	4.25	0.81	*t* = 2.07*	0.001
		Yes	3.93	1.28		
	Satisfaction	No	4.14	0.76	*t* = 7.92**	0.001
		Yes	3.05	1.06		
	Efficiency (only one visit needed)	No	3.76	0.93	*t* = 1.66	0.10
		Yes	3.50	0.86		

**p < 0.01; *p < 0.05

## Discussion

This study aimed to assess the effectiveness of the Seha app, an e-health app, in improving healthcare delivery and population health by ensuring patient satisfaction with the care given, increasing access to care, and improving efficiency in the healthcare system. The study also aimed to understand potential factors contributing to the use of the Seha app and determine whether there are technical issues affecting access, satisfaction, and efficiency. The study results provide evidence that the use of the Seha app improved the delivery of healthcare services in Saudi Arabia. Our results show that in terms of ease of access, satisfaction, and efficiency (measured in number of required doctor visits), the users of the mobile app had a better experience with health services than the users of the traditional providers. In Saudi Arabia, the national priorities for the improvement in the delivery of healthcare services are value for the money spent, quick and easy access for all, and user satisfaction. One of the major approaches for improving the delivery of healthcare in Saudi Arabia is the adoption of digital health. As the provision of healthcare through traditional methods continues to fall behind in terms of access and the quality of care, the introduction of technology and the use of mobile apps could be ways to overcome this deficiency. The findings of our current study reveal that adopting e-health technologies could address some of the persistent problems in the health system, such as limited resources, long waiting times, and general dissatisfaction with health services [11–13]. Online consultations have been found to be very effective in reducing patient expenses for the healthcare services provided [14, 25]. Our results supported this finding, as the users of the Seha app required fewer doctor visits than the users of traditional services, indicating the potential for increased adoption of the app to improve the efficiency of the health system and to reduce the pressure on traditional providers. Our findings are also consistent with previous research that discovered that e-health apps helped improve health service access and patient satisfaction [1–5, 26].

Technical problems are common in the use of mobile apps, which might create a barrier to the use of the technology [15]. For example, in our sample, 26% of the Seha app users reported having technical problems when using the app for the first time, and only 17% reported facing technical problems continuously. This finding would seem to indicate that the app was reliable most of the time. However, our results show a significant association between technical problems and lower reported satisfaction and ease of access to health services. Addressing and fixing these technical problems should help improve the overall effectiveness of the app in the delivery of healthcare. Another barrier to using mobile apps was found to be the lack of awareness about them [27], which we observed in our sample, in which 53% did not use the Seha app and as many as 43% reported not being aware of its existence. This finding points to the need for more awareness campaigns and advertisements to increase the use of the app to access health services and eventually improve the level of patient satisfaction with the app. However, 57% of the non-users in our study were aware of the app but did not use it, which might reflect findings from previous studies that revealed that people were reluctant to use mobile apps due to a lack of trust in their ability to solve health problems [19]. Improving public trust in these health technologies through education and campaigns should, therefore, help increase the utilization of the app.

Our study explored several demographic factors as well as the source of healthcare, which are known to contribute to app utilization. The findings reveal that the younger population used the mobile app more, which is consistent with previous research [28]. This is probably because the younger population is more technology savvy than the elderly population. Nevertheless, another study found that elderly patients were among the groups that were very motivated to use e-consultation services [27]. This finding would seem to suggest the importance and impact of educating the older population on the use and advantages of mobile apps to improve their overall experience with healthcare services. Gender was also identified as having a significant influence on online activities and the acceptance of health technologies [29]. In our study, we found that male participants used the mobile app more often than females, which was consistent with a previous study that observed that males had a higher level of mobile health adoption motivation than females [30]. This difference between males and females in using healthcare apps should be recognized by decision-makers involved in the development of these apps to develop strategies to overcome this gender divergence. Interestingly, the participants who lived in the western region of Saudi Arabia had a significantly lower rate of app utilization. The western region consists of the major cities and has the highest population concentration in the Kingdom. However, despite the number of major hospitals in the western region, there are few or no e-health services established for patients [31]. A study conducted in the western region found that there was inadequate parental knowledge of alternate healthcare services, such as the Seha app [20]. In our study, half of the non-users reported the emergency room as their usual source of care, which indicated the important need to develop strategies for increasing the use of e-health apps, which should help eventually reduce the non-urgent use of emergency departments.

We identified other factors that seemed to increase the likelihood of the use of the Seha app, including working in the public sector and using MoH and military health facilities as usual sources of care. In Saudi Arabia, the MoH manages 60% of the health facilities in the country, with military and private sectors covering the remaining 40%. There might be a misperception among the public that the app is only available for individuals who use MoH facilities, as the app was developed by this institution. However, the app is available for citizens and expatriates working in both the public and private sectors, so there seems to be a need to increase the public’s awareness about the availability of the app to everyone, regardless of the type of their health coverage.

This study has some limitations; for example, its cross-sectional design did not allow the establishment of causal associations between the use of e-health apps and access to, satisfaction with, and efficiency of the health system. Additionally, the study examined two groups—users and non-users of the app—to measure access, satisfaction, and efficiency, and the non-users of the app were not asked to infer how they would access health services through the app, how satisfied they were with health services access through the app, or how efficient the app was. Therefore, the findings should be interpreted with caution. However, this study provides a national exploration of the public perception of e-health apps, which should lead to further studies on the subject.

## Conclusion

This study provided evidence that the Seha app improved the delivery of healthcare in Saudi Arabia, as the findings indicated that users of the app had a better health experience in terms of their perceived ease of access to healthcare services; their satisfaction with healthcare services; and the efficiency of the system, measured by the number of required doctor visits. Other factors that appeared to influence the use of the app included age, gender, the usual source of care, and the technical difficulties experienced. Future research is recommended to explore the barriers to and motivators for using mobile health apps.

## Supplementary Information

**Additional file 1.** The additional file (supplementary file) provide title (Appendix 1) provide descriptive information of non-users health services utilization and app awareness.

## Data Availability

The dataset used was uploaded with the paper submission as a supplementary file.

## Abbreviations

MoH: Ministry of Health
IRB: Institutional Review Bord
KSU: King Saud University
