# Supplementary material for: Evaluation of e-health (Seha) application: a cross-sectional study in Saudi Arabia
Source: BMC Med Inform Decis Mak. 2021 Mar 18;21:103. doi: 10.1186/s12911-021-01437-6 (PMC7977258; doi:10.1186/s12911-021-01437-6)
Supplement: Supplementary file 1 — Additional file 1. The additional file (supplementary file) provide title (Appendix 1) provide descriptive information of non-users health services utilization and app awareness. [file 12911_2021_1437_MOESM1_ESM.docx]

**Evaluation of E-health (Seha) Application: A cross-sectional Study in Saudi Arabia**

Abeer Alharbi, Joharah Alzuwaed, Hind Qasem

**Additional file 1: Appendices**

Appendix [1] Descriptive information of non-users health services utilization and app awareness (N=279)

| **Item** | | **N** | **%** |
| --- | --- | --- | --- |
| Aware of the Seha app | No | 123 | 44.1 |
|  | Yes | 156 | 55.9 |
| Use emergency health services | No | 139 | 49.8 |
|  | Yes | 140 | 50.2 |
| Use out-patients clinics in public hospitals | No | 130 | 46.6 |
|  | Yes | 149 | 53.4 |
| Use out-patients clinics in private hospitals | No | 87 | 31.2 |
|  | Yes | 192 | 68.8 |
| Use Primary Care Centers | No | 163 | 58.4 |
|  | Yes | 116 | 41.6 |
